# Do we overlook predictive factors in Poseidon 1 patients? A retrospective analysis co-evaluating antral follicle counts & diameters

**DOI:** 10.1186/s13048-023-01323-x

**Published:** 2024-01-02

**Authors:** Gürkan Uncu, Kiper Aslan, Cihan Cakir, Berrin Avci, Isil Kasapoglu, Carlo Alviggi

**Affiliations:** 1 Faculty of Medicine, Dept. of Obstetrics and Gynecology, Bursa Uludag University, Bursa, Turkey; 2 Faculty of Medicine, Dept of Histology and Embryology, Bursa Uludag University, Bursa, Turkey; 3 Department of Neuroscience, Reproductive Science and Odontostomatology, University of Naples Federico II, Naples, Italy

**Keywords:** Ovarian Reserve, Antral Follicle, Anti-mullerian hormone, Poor ovarian response, Poseidon

## Abstract

**Background:**

An unexpected impaired ovarian response pertains to an insufficient reaction to controlled ovarian hyperstimulation. This deficient reaction is identified by a reduced count of mature follicles and retrieved oocytes during an IVF cycle, potentially diminishing the likelihood of a successful pregnancy. This research seeks to examine whether the characteristics of antral follicles can serve as predictive indicators for the unexpected impaired ovarian response to controlled ovarian stimulation (COS).

**Methods:**

This retrospective cohort study was conducted at a tertiary university hospital. The electronic database of the ART (assisted reproductive technologies) center was screened between the years 2012–2022. Infertile women under 35 years, with normal ovarian reserve [anti-Müllerian hormone (AMH) > 1.2 ng/ml, antral follicle count (AFC) > 5] who underwent their first controlled ovarian stimulation (COS) cycle were selected. Women with < 9 oocytes retrieved (group 1 of the Poseidon classification) constituted the group A, whereas those with ≥ 9 oocytes severed as control (normo-responders) one (group B). Demographic, anthropometric and hormonal variables together with COS parameters of the two groups were compared.

**Results:**

The number of patients with < 9 oocytes (group A) was 404, and those with ≥ 9 oocytes were 602 (group B). The mean age of the group A was significantly higher (30.1 + 2.9 vs. 29.4 + 2.9, *p* = 0.01). Group A displayed lower AMH and AFC [with interquartile ranges (IQR); AMH 1.6 ng/ml (1-2.6) vs. 3.5 ng/ml (2.2–5.4) *p* < 0.01, AFC 8 (6–12) vs. 12 (9–17), *p* < 0.01]. The number of small antral follicles (2–5 mm) of the group A was significantly lower [6 (4–8) vs. 8 (6–12) *p* < 0.01), while the larger follicles (5–10 mm) remained similar [3 (1–5) vs. 3(1–6) *p* = 0.3] between the groups.

**Conclusion:**

The propensity of low ovarian reserve and higher age are the main risk factors for the impaired ovarian response. The proportion of the small antral follicles may be a predictive factor for ovarian response to prevent unexpected poor results.

## Background

Despite the advanced technology in assisted reproductive techniques (ART) for years, it is still impossible to predict accurately the ovarian response in patients who undergo controlled ovarian stimulation (COS). Clinicians may still face poor ovarian response despite doing accordance with evidence algorithms. In this case, explaining why a poor response is obtained in a young patient with a normal ovarian reserve and who has been stimulated with the appropriate dose of gonadotropin can be challenging. This situation is called “unexpected poor ovarian response” (uPOR). The incidence of uPOR varies between 10 and 40% depending on the threshold of the collected oocytes (4 or 9) [1–3]. While the Bologna criteria are the most accepted among these poor ovarian response classifications, the POSEIDON classification has been published recently and used worldwide [4–6]. The Poseidon classification is consistent with data reported by Drakopoulos et al. [1] which identified different prognostic categories in terms of live birth rate according to the number of oocytes retrieved. These data described an oocyte retrieval between 10 and 15 as “optimal”, while a number ranging between 4 and 9 as “suboptimal”. Finally, a number of oocytes between 1 and 3 was considered as “poor”. According to the POSEIDON criteria, the patients who have a number of oocytes retrieved between 1 and 9 despite normal ovarian reserve [antral follicle count (AFC) > 5–7, anti-Müllerian hormone (AMH) > 1.2 ng/ml] represent the unexpected impaired ovarian responses (uIOR) and are classified as groups 1 and 2. More specifically, women younger than 35 years of age are classified as POSEIDON Group-1 whereas, among them, those having 1–3 and 4–9 oocytes retrieved are identified as subgroups 1a and 1b, respectively. On the other hand, women aged 35 years and older with similar outcomes are categorized as POSEIDON Group 2a and 2b. Recent evidence confirmed that Poseidon groups 1 and 2 have significantly lower live birth rate (LBR) when compared with women having more than 9 oocytes retrieved, confirming the capability of Poseidon classification to identifying different low prognosis segments [7]. The exact etiology of uIOR or POSEIDON-1 remains unclear. There are various blamed factors for this phenomenon, including polymorphisms of gonadotropins and their receptors, dietary habits, environmental variable and drug administration errors [8–10]. On the other hand, novel developed indexes like follicle output rate (FORT - The ratio the number of pre-ovulatory follicles (16–22 mm in diameter) x 100 to the number of pre-antral follicles (3–8 mm in diameter)) and follicle to oocyte index (FOI- The ratio of the total number of oocytes collected at the end of stimulation to the number of antral follicles available at the start of stimulation) aim to assess intracycle ovarian response [11, 12] before oocyte pick up. However, none of these etiologies could be predicted before COS, and none of the developed methods could avoid uIOR. Therefore, studies mainly focused on what can be done after uIOR to prevent recurrent POR.

The present study aims to investigate any patient characteristics that could predict and avoid impaired ovarian response (Poseidon 1 profile) before COS.

## Methods

### Study design & ethical approval

This retrospective cohort study was conducted at a tertiary university hospital’s Assisted Reproductive Technologies (ART) center. The study protocol was approved by the clinical trials ethical committee of the university with the number 2023-13/13.

### Patient selection

The ART center’s electronic database was screened between 2012 and 2022. Patients who were eligible for POSEIDON-1 criteria (age < 35 years, AMH > 1.2 ng/ml, AFC > 5) were selected from all COS cycles. Patients who underwent their first COS cycle were enrolled to avoid bias. Patients with body mass index (BMI) over 35 kg/m2 and oocyte cryopreservation cycles due to any malignity were excluded from the study.

### Assessment of ovarian reserve

#### Antral follicle count

In all patients, the evaluation of antral follicle count was conducted through transvaginal ultrasound. The follicular assessment, as per Broekmans’ recommendations [13], took place just before ovarian stimulation on the 2nd or 3rd day of the menstrual cycle. The entire ovaries underwent scanning, and follicles within the 2–9 mm range were tallied. Antral follicles were categorized into two subgroups based on their size: those between 2 and 5 mm were designated as AFCa, while follicles ranging from 5 to 10 mm were labeled as AFCb.

#### Anti-mullerian hormone

Blood samples were collected on any day of the menstrual cycle and Anti-Mullerian hormone was analyzed by “Beckman Coulter Access II” enzymatic-immunoassay. The detection limit of the test was ≤ 0.02 ng/mL.

### COS-ICSI-ET protocol

The routine infertility work-up was applied to all patients. After the basal transvaginal ultrasound check, the COS was started on the second or third day of the menstrual cycle. The daily gonadotropin dosage was adjusted depending on the patient’s age, BMI, ovarian reserve, and previous COS history. Recombinant Follicle Stimulating Hormone (rFSH) was used for COS. The standard COS protocol was the flexible antagonist protocol with hCG trigger. The trigger was applied when at least three follicles reached 17–18 mm.

### Interventions

Patients were divided into two groups. All the results were compared between these groups. Group A consisted of patients with lower than nine oocytes, and Group B patients with equal or higher than nine oocytes. Group A was divided in two subgroups as Poseidon 1a (< 4 oocytes) and Poseidon 1b (4–9 oocytes). Demographic parameters (age, BMI, infertility etiology, ovarian reserve tests (AMH, AFC, number of small antral follicles (AFC-a, 2–5 mm), number of large antral follicles (AFC-b, 5–10 mm)) and COS cycle parameters were recorded.

### Statistical analysis

Continuous variables are defined as mean ± standard deviation (SD) or median (with 25th-75th percentiles- IQR) depending on the distribution. Categorical variables are defined as percentages. As appropriate, continuous variables were compared between the groups using the independent samples t-test or the Mann-Whitney U test. Categorical variables were compared using the Chi-square test and its derivatives. Binary logistic regression analysis was performed to assess the association of variables with Poor ovarian response. Partial correlation analysis was also performed to study the linear relationship between variables after excluding the effect of independent factors. A two-sided *p*-value of 0.05 was considered statistically significant.

## Results

There were 7458 COS cycles between 2012 and 2022. A total of 1006 patients were enrolled in. There were 404 patients in Group A (uIOR group) and 602 patients in Group B (normo-responder – NOR group). There were 80 patients in Poseidon 1a group and 324 patients in Poseidon 1b group.

**Fig. 1:**
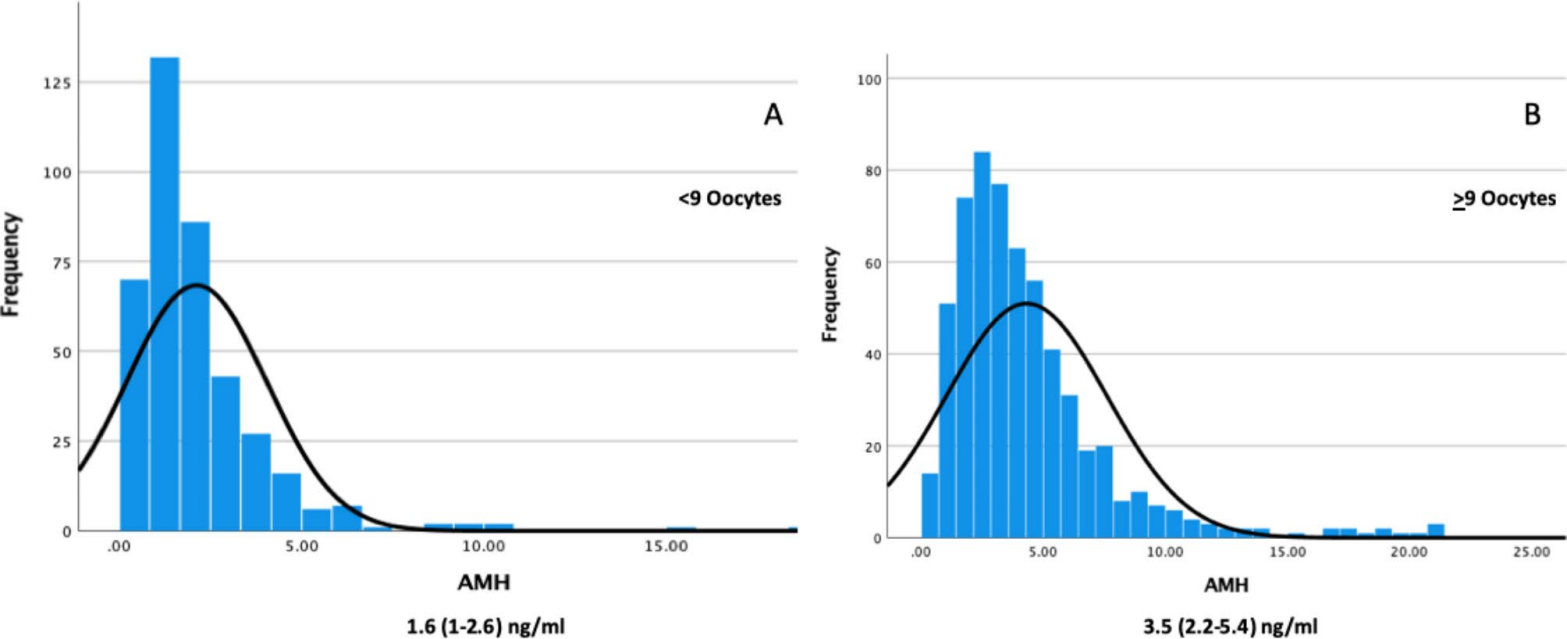
AMH Histograms of the groups (Figure **A** refers to patients with < 9 oocytes and Figure **B** refers to patients with ≥ 9 oocytes)

The mean age was significantly higher in Group A than in the other group (30.1 + 2.9 vs. 29.4 + 2.9, *p* = 0.01). The infertility etiologies were similar between the groups. Although all included patients had a normal ovarian reserve, Group-A had significantly lower AMH and AFC than Group-B [Respectively, with IQR; AMH 1.6 ng/ml (1-2.6) vs. 3.5 ng/ml (2.2–5.4) *p* < 0.01, AFC 8 (6–12) vs. 12 (9–17), *p* < 0.01 (Figure-1)]. When the AFC was further analyzed, the proportion of the small antral follicles (AFC-a, 2–5 mm) was lower in Group-A, while the proportion of larger antral follicles (AFC-b, 5–10 mm) remained similar between the groups. The mean daily gonadotropin dosage was significantly higher in Group-A (302 + 64 IU vs. 250 + 67 IU *p* < 0.01).

The collected oocyte numbers (with IQR; 6 (4–8) vs. 15 (12–20) *p* < 0.01), metaphase-2 (MII) oocyte numbers (4 (2–6) vs.12 (9–16), *p* < 0.01) and two-pronuclei (2PN) embryo numbers [2 (1–4) vs. 7 (5–10), *p* < 0.01] were significantly lower in Group-A (Table-1).

The results did not change when the Group-B was further compared with Poseidon subgroups 1a and 1b. It was revealed that Poseidon 1a has significantly lower ovarian reserve and higher age than Poseidon 1b and the control group. However, AFC-b was still similar in each group (Table-2).

Binary logistic regression analysis assessed the association of significant factors on ovarian response. Only AMH and AFC-a showed significance (Table-3). A partial correlation analysis was further performed between AFC-a and impaired ovarian response, excluding the effect of AMH, and the AFC-a showed a positive correlation with ovarian response. The increasing number of small antral follicles is associated with better ovarian response (Table-4).

## Discussion

The present study showed that characteristics of antral follicles at the moment of AFC may help to predict impaired ovarian response (IOR), including both poor and suboptimal profiles, even in women < 35 years of age (POSEIDON groups 1a and 1b). In particular, our results indicated that, even in presence of an apparently normal AFC, the proportion of small antral follicles (AFC-a, 2–5 mm) may be an indicator in predicting IOR in detailed evaluation. The propensity of low ovarian reserve and higher age also emerges as main risk factors for uIOR and POSEIDON 1 profile.

Besides the age and ovarian reserve, numerous factors such as the consumption of high glycemic index foods [8], the presence of air pollution in the living area [14], and exposure to substances like benzene raise the risk of uIOR [15]. Despite studies showed higher concentrations of these substances in the follicle fluids of patients with unexpected poor ovarian response, it is not feasible to accurately assess the dietary habits or exposure to harmful substances and predict uIOR in women with normal ovarian reserve.

Beyond these possible etiologies, the most causatively asserted one is FSH receptor (FSHR) polymorphisms. Since the 90s, many studies have confirmed that FSHR polymorphisms might increase gonadotropin consumption and unexpected poor ovarian response [10]. Till today, various polymorphisms have been shown to affect ovarian sensitivity negatively. A meta-analysis that involves studies about different FSH & LH receptor genotypes and ovarian sensitivity identified that polymorphisms could untangle this issue in clinical practice [16]. However, the cost/effectiveness evaluation of screening all the population for FSHR receptor polymorphism to personalize the gonadotropin dose is still matter of debate. Furthermore, a recently published randomized controlled trial showed that the presence of FSHR polymorphism did not negatively affect FORT and FOI scores of the patients and concluded that genotyping the FSHR prior to COS in normo-responder patients should not be routinely recommended [17].

Another possible etiology that causes uIOR may be incorrect drug administration or insufficient patient education by ART nurses. Despite accurately calculated gonadotropin dosage by ART physicians, patients may inject lower doses of daily gonadotropin or misuse it. Therefore, the main reason for this situation may be inappropriate drug applications that we never anticipated. A multicenter survey study in France reported that almost 20% of the patients misunderstand the drug doses, COS schedule, or application way [9]. Thus, this possibility should always be kept in mind.

Despite the above-listed possible etiologies for uIOR, predicting both poor and suboptimal profiles in women with normal ovarian reserve is still tricky. The management strategies in the presence of uIOR are mainly based on gonadotropin dose or type adjustment. Firstly, gonadotropin dose increment in the same cycle may be an option when unexpected initial uIOR is observed. De Placido et al. showed that dose increment with recombinant LH might be an effective option for ovarian outcome in patients with an initial inadequate ovarian response to rFSH alone [18]. On the other hand, dose adjustment in the subsequent cycle may be beneficial in uIOR patients. Drakopoulos et al. reported that an increase of 50 IU of the initial rFSH dose would lead to 1 more oocyte in the subsequent cycle of patients with uIOR [2]. another COS option in this group of patients is dual stimulation [19]. As is known, follicular recruitment is an ongoing condition and involves mainly three follicular waves in the one-month cycle [20]. Therefore, each follicular wave may show fluctuations in antral follicle count, and COS outcome may change cycle by cycle. Eftekhar et al. [21] studied dual stimulation in patients with uIOR. They reported that the number of collected oocytes was higher in luteal phase stimulation than the follicular phase stimulation in patients with uIOR (Respectively; mean oocyte number; 9.2 + 6.8 vs. 1.9 + 1.1). Cimadomo et al. [22] also reported that luteal phase stimulation of the same cycle provides more blastocyst in poor prognosis patients.

Although there are various management strategies for patients with a history of uIOR, the main issue is to predict this situation before it happens. The present study aimed to investigate if there is any overlooked predictive factor and results indicated that the proportion of small antral follicles gains importance in predicting the ovarian response. According to our results, a decrease in small antral follicle count causes low FORT and FOI scores. The lower proportion of small antral follicle count (sAFC) is related to ovarian hyposensitivity. Thus, it may be concluded that the proportion of small antral follicles shows functional ovarian reserve. This hypothesis was mainly mentioned in the 90s by different authors. Faddy et al. [23] showed a biphasic pattern with a steeper decline of follicles after the age of 37.5 years. Scheffer et al. [24] showed a steeper yearly decline in the number of small antral follicles (2–5 mm) than for larger follicles (6–10 mm). Haadsma et al. [25] reported that the number of small antral follicles (2–6 mm) is significantly related to age and also, independent of age, to all ovarian reserve tests, suggesting the number of small antral follicles represents the functional ovarian reserve.

Similar to the above-published studies, we previously studied small antral follicle count and its clinical implications in ART. We showed that a high ratio of small-size (2–5 mm) basal antral follicles is a predictive factor for higher ovarian response to ovarian hyperstimulation [26]. We also demonstrated that between the patients with similar AFC, sAFC proportion determines the AMH discordance independent from age [27]. As is known, AMH is mainly produced by pre-antral and smaller antral follicles up to 4–6 mm, and an increase in proportion of sAFC provides a higher AMH level [28]. However, the impact of the increased proportion of small antral follicles (sAFC) on not just the quantity but also the quality of oocytes remains uncertain. Our findings raise the question of whether there is a correlated increase in the rate of immature oocytes associated with the proportion of sAFC. Despite lacking morphological outcomes of the oocytes, our results align with the Maribor criteria, evaluating embryological factors such as the metaphase oocyte rate, fertilization rate, and blastulation rate [29], demonstrating comparable outcomes.

Our study has some limitations. The retrospective design limits the power of the results. Including only the first COS cycle of each patient, a relatively high number of the groups, and the presence of regression analysis to confirm the significant factors strengthen our findings.

In the realm of clinical practice, the outcomes of our study shed light on the significance of antral follicle characteristics at the time of antral follicle count (AFC) in predicting impaired ovarian response (IOR) among women, even those under the age of 35 (POSEIDON groups 1a and 1b). Notably, our results highlight that, despite an apparently normal AFC, the proportion of small antral follicles (AFC-a, 2–5 mm) may serve as an indicator for predicting IOR during detailed evaluation. The identification of risk factors such as low ovarian reserve and advanced age further underscores the relevance of our findings, offering valuable insights for clinical decision-making.

Looking ahead, future research endeavors should explore integrated predictive models that encompass variables such as the proportion of small antral follicles, age, and AMH levels near the threshold before Controlled Ovarian Stimulation (COS). The combination of these factors has the potential to enhance our ability to forecast ovarian response, aiding in the development of personalized and more effective management strategies. Moreover, additional randomized controlled trials are warranted to validate and refine the insights gleaned from our study, ultimately contributing to the ongoing evolution of tailored approaches for individuals facing impaired ovarian response. The unpredictable nature of uPOR emphasizes the ongoing need for innovative research that can bring us closer to unraveling its complexities and improving outcomes for both patients and clinicians.

**Table 1:** Demographic Parameters and Cycle Outcomes of the Groups

	< 9 Oocytes (N = 404)	≥ 9 Oocytes (N = 602)	*p*
**Age (yrs.)**	30.1 ± 2.9	29.4 ± 2.9	0.01
**BMI (kg/m** ^**2**^ **)**	24.5 ± 4.7	24.9 ± 4.2	0.06
**Etiology** **Unexplained** **Tubal** **Anovulation** **Male** **Both** **Others**	29.6% 4% 12% 28% 10% 9.4%	30% 6% 14% 25% 11% 14%	0.4
**FSH (IU/L)**	5.4 (4-6.8)	5 (3.8–6.1)	< 0.01
**Estradiol (ng/ml)**	48 (32–87)	43 (31–68)	0.09
**AMH (ng/ml)**	1.6 (1-2.6)	3.5 (2.2–5.4)	< 0.01
**AFC (n)**	8 (6–12)	12 (9–17)	< 0.01
**AFC – a**	6 (4–8)	8 (6–12)	< 0.01
**AFC – b**	3 (1–5)	3 (1–6)	0.3
**Daily Gonadotropin Dose**	302 ± 64	250 ± 67	< 0.01
**Estradiol on hCG day**	1030 (670–1644)	2292 (1502–3368)	< 0.01
**No. of Oocytes**	6 (4–8)	15 (12–20)	< 0.01
**Metaphase-2 Oocytes**	4 (2–6)	12 (9–16)	< 0.01
**No. of 2-Pronuclei Embryos**	2 (1–4)	7 (5–10)	< 0.01

*Mean values with standard deviation, median values with quartiles

**Table 2:** Demographic Parameters and Cycle Outcomes of the Poseidon Subgroups (1a and 1b)

	< 4 (N = 80)	4–9 Oocytes (N = 324)	> 9 Oocytes (N = 602)	*p*
**Age**	**30.7** **±** **2.7**	29.9 ± 3.0	**29.4** **±** **2.9**	**0.01**
**BMI (kg/m** ^**2**^ **)**	24.3 ± 4.8	24.6 ± 4.7	24.9 ± 4.2	0.07
**FSH (IU/L)**	**5.7 (3.7–7.9)**	5.4 (4.1–6.7)	**5 (3.8–6.1)**	**< 0.01**
**Estradiol (ng/ml)**	58 (36–110)	47 (31–83)	43 (31–68)	0.02
**AMH (ng/ml)**	**1.4 (1.2–2.1)**	1.8 (1.3–2.8)	**3.5 (2.2–5.4)**	**< 0.01**
**AFC (n)**	**7 (6–9)**	9 (7–12)	**12 (9–17)**	**< 0.01**
**AFC – a**	**5 (3–7)**	6 (4–8)	**8 (6–12)**	**< 0.01**
**AFC – b**	2 (1–4)	3 (1–5)	3 (1–6)	0.143
**Daily Gonadotropin Dose**	300 ± 55	300 ± 66	250 ± 67	< 0.01
**Estradiol on hCG day**	690 (341–1243)	1148 (752–1740)	2292 (1502–3368)	< 0.01
**No. of Oocytes**	2 (1–3)	7 (5–8)	15 (12–20)	< 0.01
**Metaphase-2 Oocytes**	1 (0–2)	5 (4–7)	12 (9–16)	< 0.01
**No. of 2-Pronuclei Embryos**	1 (0–1)	3 (1–4)	7 (5–10)	< 0.01

*Mean values with standard deviation, median values with quartiles

**Table 3:** Logistic Regression Analysis

Logistic Regression Analysis						
	B	S.E.	Wald	df	Sig.	Exp(B)
Age	.003	.042	.004	1	.951	1.003
BMI	− .014	.029	.241	1	.623	.986
Etiology			5.197	8	.736	
FSH	− .061	.041	2.138	1	.144	.941
**AMH**	**.408**	**.105**	**15.013**	**1**	**< .001**	**1.503**
AFC	− .006	.035	.027	1	.869	.994
**AFC-a**	**.143**	**.043**	**11.224**	**1**	**< .001**	**1.154**
Gonadotropin Dose	− .005	.003	2.949	1	.086	.995
Constant	.674	1.630	.171	1	.679	1.961

**Table 4:** Partial Correlation Analysis

Partial Correlations				
Control Variables			AFC-a	Poseidon1
AMH	**AFC-a**	Correlation	1.000	0.223
		Significance (2-tailed)	.	**< 0.001**
		df	0	492
	Poseidon1	Correlation	0.223	1.000
		Significance (2-tailed)	**< 0.001**	.
		df	492	0

## Data Availability

All data generated or analyzed during this study are included in this published article and are available from the corresponding author on reasonable request.

## Declarations

### Attestation statements

Data regarding any of the subjects in the study has not been previously published unless specified.
